# Somatic Markers, Rhetoric, and Post-truth

**DOI:** 10.3389/fpsyg.2017.01273

**Published:** 2017-07-26

**Authors:** José M. Muñoz

**Affiliations:** External Research Collaborator at the Mind-Brain Group, Institute for Culture and Society, University of Navarra Pamplona, Spain

**Keywords:** emotions, somatic markers, decision-making processes, mental states, Aristotle, post-truth

Emotions play an important role in many volitional processes. In this regard, the somatic marker hypothesis (SMH) (Damasio et al., 1991; Damasio, 1994, 1996) has proven enormously influential in cognitive science. Despite having been formulated more than two decades ago, much related bibliography has appeared in recent years (e.g., Leland and Grafman, 2005; Reimann and Bechara, 2010; Olsen et al., 2015). In this paper, firstly, I will reflect on how the SMH contributes to an integrative conception of the human body and mind from a neurophilosophical perspective. Secondly, I will propose an example that suggests that somatic markers play an important role in so-called “post-truth politics.”

## Somatic markers as mental states

A brief description of the SMH is in order. According to Antonio Damasio, in his book *Descartes' Error* (Damasio, 1994), when faced with conflicting decisions a person will usually respond by imagining a series of hypothetical scenarios, represented by images which follow rapidly one after another. It is possible to resolve the conflict by proceeding in a purely rational way, studying the advantages and drawbacks of each of the scenarios. However, this entails a complex calculation that depends largely on the production of new hypothetical scenarios and the verbal narratives associated with them. There are various difficulties with this model, including the need for our memory to keep all necessary information during the entire process of reasoning. But in fact we are able to make decisions in short spaces of time, which implies that something more than reason must be involved.

Suppose that, prior to the reasoning process, you feel a brief, unpleasant sensation when imagining a negative consequence to a possible decision. You are then experiencing a somatic marker, that is, a bodily sensation that is associated with a scenario imagined by an agent. According to Damasio, “*somatic markers are a special instance of feelings generated from secondary emotions*. Those emotions and feelings *have been connected, by learning, to predicted future outcomes of certain scenarios*” (Damasio, 1994). Somatic markers, which are managed by the prefrontal cortex and able to act consciously or unconsciously, operate as *assistants in decision-making processes*, because they can immediately lead us to dismiss, or to consider, one option versus other alternatives. They can be negative or positive, depending on whether they act as alarm signals or as incentives, respectively. Yet, they are not necessarily sufficient for decision-making in all situations, since in many cases they make it simpler for later reasoning to be carried out^1^. At times somatic markers may also hinder reasoning.

In my view, the SMH provides valuable tools for a unitary understanding of the human mind and body. For instance, consider a four-way typology of mental states and properties (Moya, 2006): intentional states (intentions, desires, beliefs, etc.), phenomenological states (sensory experiences, pleasure, pain…), mixed states (emotions and feelings), and pure dispositions (e.g., intelligence, envy, generosity). The third category combines intentional and phenomenological traits: mixed states are “characterized both by a certain attitude towards a content and by a certain felt quality”; furthermore, the “phenomenological component is nonspecific when it is isolated from the content” (Moya, 2006). This seems to be the case with somatic markers. As Damasio says, they consist of bodily sensations linked to possible consequences internally projected by the agent in the face of hypothetical scenarios. Suppose, for example, that the agent experiences an unpleasant sensation every time she imagines the negative consequences of a given decision. What we have here is a felt quality (the unpleasant sensation) which, far from being nonspecific or vague, points to a certain mental content (the decision); it thus turns out to be an attitude (rejection) at the same time^2^. Hence, employing the typology given above, somatic markers should be classified as mixed mental states, with both intentional and phenomenological features.

## Somatic markers as aristotelian emotions

Furthermore, there is a similarity between the SMH and Aristotle's conception of emotions in his *Rhetoric* (cf. Sifakis, 2001). He “considers the passions or emotions to be psychophysical affections, associated with physiological alterations, and which involve sensations of pain and/or pleasure” (Trueba, 2009). A first parallel can be drawn here, insofar as somatic markers consist of pleasant or unpleasant sensations (i.e., pleasure and pain) originating in our body and associated with mentally projected images (the psychophysical connection). But the *functions* which the Macedonian thinker assigns to pain (or grief) and pleasure endow the SMH with an even stronger Aristotelian “halo”: “The emotions [*pathē*]^3^ are those things through which, by undergoing change, *people come to differ in their judgments* [italics added] and which are accompanied by pain and pleasure, for example, anger, pity, fear, and other such things and their opposites” (Rhetoric, II, 1, 1378a 20; Aristotle, 2007). Like somatic markers, the emotions in *Rhetoric* enjoy the power to influence rational processes, which they do precisely in virtue of the pleasantness or unpleasantness of their physical component. Nevertheless, here they do not have the moral connotation that they do in other Aristotelian treatises. In the *Nicomachean Ethics*, for example, he argues that “if the virtues are to do with actions and *situations of being affected* [i.e., passions; italics added], and pleasure and pain follow from every action and situation of being affected, then this is another reason why virtue will be concerned with pleasures and pains” (Nicomachean Ethics, II, 3, 1104b 15; Aristotle, 2000). But Damasio does not focus on the moral aspect of emotions, so the similarity between his hypothesis and Aristotle's conception is more remarkable in the case of *Rhetoric* than in others of his works.

Perhaps the most important idea that Damasio defends in *Descartes' Error* is that the brain does not act as a solitary agent isolated from the rest of the body (Damasio, 1994). To affirm the contrary would be to fall into what has been eloquently described as “a sort of materialistic dualism” (Moya, 2011): “Whereas, according to old forms of dualism, a human being is essentially a soul or thinking thing that contingently inhabits a body, for this new dualism a human being is essentially a brain that contingently inhabits (the rest of) the body. In current philosophy of mind, and even epistemology, the importance of the brain, which we do not want to deny, has been magnified, with a corresponding devaluation of other parts of the human body, such as the tongue or the hands, which tend to appear as mere peripheral appendices at the service of the brain, and ultimately dispensable.” The SMH, I claim, constitutes a powerful attack against this materialistic dualism. On the one hand, it conceives of intentional traits (attitudes toward an imagined content) and phenomenological traits (felt qualities with a bodily origin) in an intermixed, inseparable way. On the other, and following Aristotle in the *Rhetoric*, it understands emotions (somatic markers) as psychophysical processes in which the rational component is influenced by the bodily component (in virtue of the pain or pleasure associated with the latter). Thus, the SMH provides an integrative view in which soma and psyche are inextricably linked. In addition, this unitary idea of the human seems to be endorsed by important empirical evidence. For example, it is has been known for more than three decades that the nervous, endocrine, and immune systems are interconnected through a network formed by neuropeptides (which act as messenger molecules) and their corresponding receptors, and that the latter are spread throughout this network in such a way that we can find them also in the brain, including regions associated with emotions (Pert et al., 1985). This reinforces the SMH, since it suggests that the nervous system is sensitive to emotional signals originating from other biological systems.

## Somatic markers as political instruments

But the implications of the SMH do not just reach the individual level. Somatic markers may in fact be relevant in political speech. More concretely, I think that they can be employed in post-truth politics, where factual truth is replaced by an appeal to personal emotions when citizens make decisions. Indeed, in several countries with well-established democratic values certain mass media may have created, through somatic markers, a collective emotional basis with undesirable political consequences.

Consider the following example. Suppose that an individual *x* is a citizen of democratic country *A*. Since her childhood, *x* has been educated in the values of compassion and empathy toward the suffering of others, which conforms to a well-established moral norm in *A*. Now imagine that, for decades, the presence or abundance of citizens from country *B* has been (voluntarily or involuntarily) associated with images of misery and violence in a great part of *A*'s mass media. Due to this, and after having watched dozens of productions with these characteristics, *x* has learned to respond to the scenario “presence/abundance of *B*-citizens” with a feeling of fear and an attitude of rejection. A negative somatic marker is thus formed. This alarm signal remains latent most of the time. However, suppose that at the right time a citizen *y* who aspires to govern *A* through the support of *x* publicly proposes measures to avoid the supposed horrible consequences associated with the scenario “presence/abundance of *B*-citizens.” In this way *y* is able to reactivate and strengthen the somatic marker “fear and rejection” in *x*. This *argumentum ad passiones* could persuade *x* to vote for *y*.

But let's go a little further. Citizen *x* might also think that avoiding the scenario mentioned will cause suffering to *B*-citizens. And let us remember that *x* has the deeply rooted values of compassion and empathy toward others' suffering. Based on these moral values, *x* might intend not to vote for *y*. As a result, *x* suffers great mental tension because of the strong cognitive dissonance resulting from the coexistence of two completely opposite intentions: to vote for *y* and not to vote for *y*. As is well known, a strong cognitive dissonance strongly motivates people to try to reduce this psychological tension (Festinger, 1957). At this point, *x* might be enormously receptive to accepting arguments from *y* in favor of avoiding the scenario “presence/abundance of *B*-citizens,” even if the facts on which those arguments are based are false. Thus, *x* would end up making a decision coherent (i.e., consonant) with her “fear and rejection” somatic marker: to vote for *y* to govern *A*.

Of course, the rhetorical strategy of the candidate *y* is far removed from the ideas of Aristotle in the *Nicomachean Ethics*, where, as we have seen, he does not admit of emotions that are unrelated to virtue.

## Author contributions

The author confirms being the sole contributor of this work and approved it for publication.

### Conflict of interest statement

The author declares that the research was conducted in the absence of any commercial or financial relationships that could be construed as a potential conflict of interest.

## Footnotes

^1^By allowing for the speedy removal of certain possibilities and the strengthening of others, somatic markers provide the agent with a manageable set of alternatives. As a result, the SMH seems to offer a solution to the frame problem when applied to human decisions (Megill and Cogburn, 2005). Although initially posed in the field of artificial intelligence (McCarthy and Hayes, 1969), this problem challenges us to explain how *intelligence* (including human intelligence) can quickly and efficiently access the needed significant information when making a decision, without having to analyze all the information that it actually possesses.

^2^I think that a very similar argument could easily be made in the case of positive somatic markers.

^3^Word and square brackets shown as in the original source.
